# A Case of Multiple Self-Involuting, Mixed Presentation, Giant Congenital Juvenile Xanthogranuloma

**DOI:** 10.7759/cureus.37644

**Published:** 2023-04-16

**Authors:** Erika Malana, Paul Kowalski, Michelle Gallagher

**Affiliations:** 1 Pediatric Dermatology, Michigan State University College of Osteopathic Medicine, East Lansing, USA; 2 Pathology, Michigan Pathology Specialists PC - Spectrum Health, Grand Rapids, USA

**Keywords:** non-langerhans cell histiocytosis, pediatric dermatology, pediatric tumors, newborn diseases, juvenile xanthogranuloma

## Abstract

Juvenile xanthogranuloma (JXG) is an uncommon benign skin disorder of infants and young children characterized by dermal proliferation and infiltration of dendrocytes. We present a unique case of giant congenital JXG with a mixed presentation of macules, papules, nodules, and ulcerations in a neonatal male who was observed until the age of 23 months, by which time all lesions had spontaneously self-involuted. Prior to complete resolution, some lesions took the form of pedunculated protrusions. To our knowledge, this is the first of this atypical case to be presented in the literature.

## Introduction

Juvenile xanthogranuloma (JXG) is a benign proliferative disorder of dermal dendrocytes and is the most common of the non-Langerhans cells histio-cytosis [1]. While etiology and incidence are unknown, a large tumor registry spanning 35 years documented 129 cases of JXG out of 24,600 pediatric tumors (0.5%) [2]. However, the frequency is likely underestimated since the diagnosis is often made clinically without routine histologic examination.

JXG typically presents in early childhood as a solitary lesion on the head, neck, and upper trunk that spontaneously resolves on its own after a few years [1]. Rarely, it can present as multiple lesions, giant lesions, or with extracutaneous involvement. 

## Case presentation

An infant boy was born at term via an uncomplicated vaginal delivery. Physical exam at birth revealed an alert neonate with right-sided scalp swelling and rash. Vital signs were stable and the remainder of the physical exam was non-contributory. The patient was transferred to the neonatal intensive care unit (NICU) with the admitting diagnosis of encephalocele. Head ultrasound and MRI demonstrated hemorrhage into deep subcutaneous tissues without evidence of an underlying cystic hygroma, consistent with a subgaleal hematoma. Abdominal ultrasound showed a normal liver and spleen while ophthalmology consult revealed no ocular involvement.

**Figure 1:**
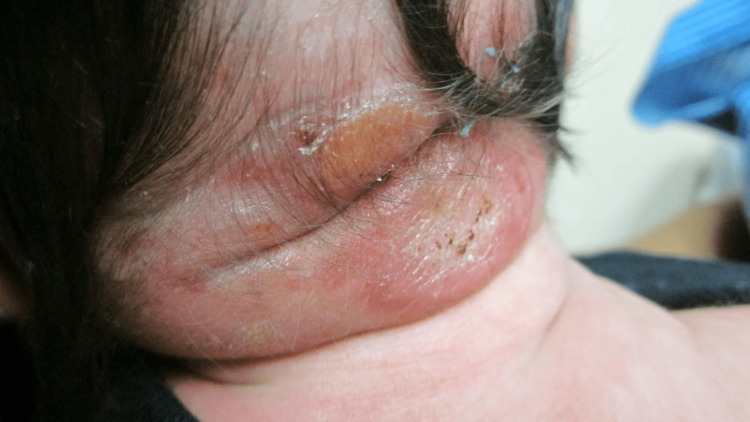
Multiple firm, mobile, non-tender subcutaneous papules and nodules measuring 1-2 cm in diameter with some erosions, scabbing and overlying erythema on the occipital scalp on day 4 of life; underlying 2.5-3 cm non-tender, hairless swelling of the right parieto-occipital scalp extending down the posterior neck due to subgaleal hematoma.

**Figure 2:**
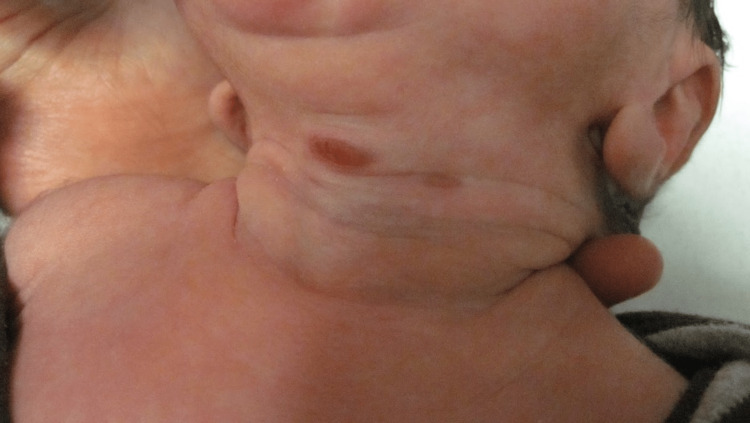
Reddish-brown macules on the anterior neck on day four of life; central lesion measured 1 cm across.

**Figure 3:**
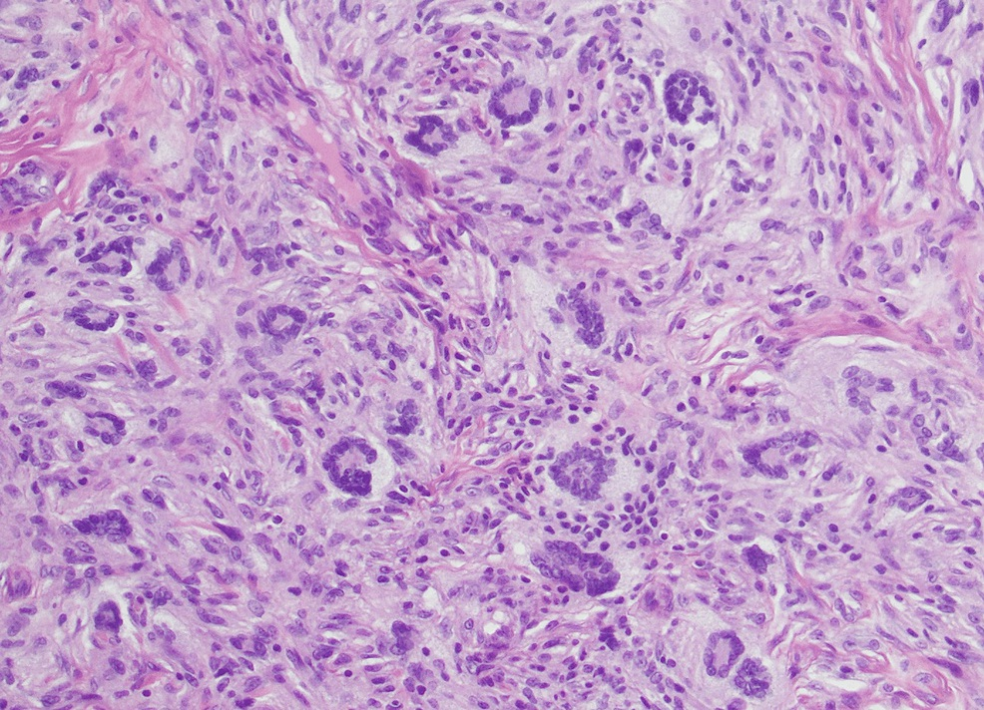
Occipital scalp biopsy revealing dermal infiltrate of uniform histiocytes with scattered Touton giant cells consistent with juvenile xanthogranuloma. Touton giant cells describe a wreath of nuclei with a homogenous eosinophilic cytoplasmic center, surrounded by foamy xanthomatization in the periphery.

Dermatology consult on day four revealed multiple firm, mobile, non-tender papules and subcutaneous nodules with overlying erythema and central erosions on the occiput of the scalp (Figure 1) with few satellite lesions throughout the scalp, jaw, and back. The papules and nodules measured 1-2 cm in diameter. However, a 2.5 cm x 3 cm hairless area of swelling of the right parieto-occipital scalp consistent with a subgaleal hematoma (Figure 1), made it difficult to determine if larger lesions were present. Additionally, two reddish-brown macules were present on the anterior neck, the larger of the two measuring 1 cm across (Figure 2). A 4 mm punch biopsy of the occiput was taken with the differential diagnoses of myofibroma, sarcoidosis, lymphoma cutis, and JXG. The patient remained stable by day five and was discharged from the NICU with instructions to follow-up with dermatology outpatient. Pathology report of the dermal stroma revealed diffuse distribution of uniform histiocytes with scattered multinucleated Touton giant cells, confirming the diagnosis of giant congenital JXG (Figure 3).

**Figure 4:**
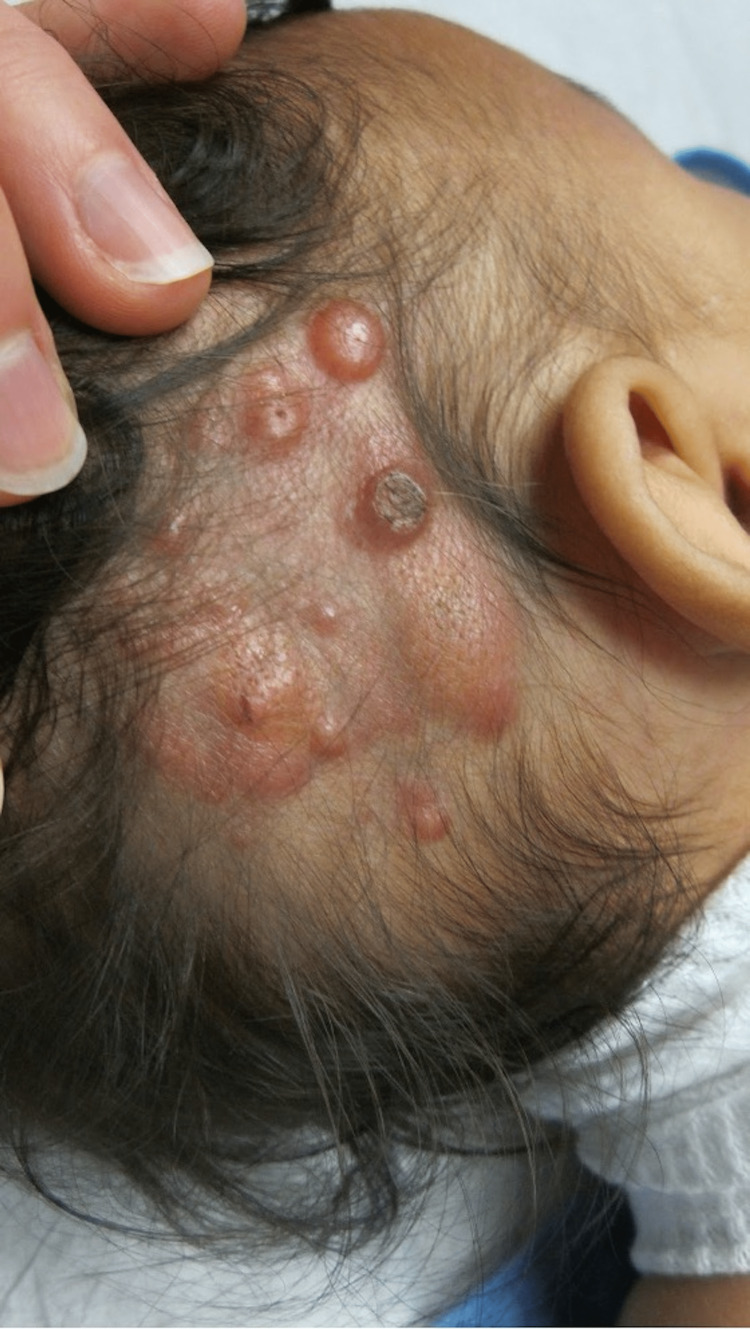
Multiple erythematous papules and nodules with and without central ulcerations, ranging in size from 1-4.5 cm in diameter, on a hairless region of the right parietal scalp consistent with giant juvenile xanthogranuloma in a neonate at four months of life.

**Figure 5:**
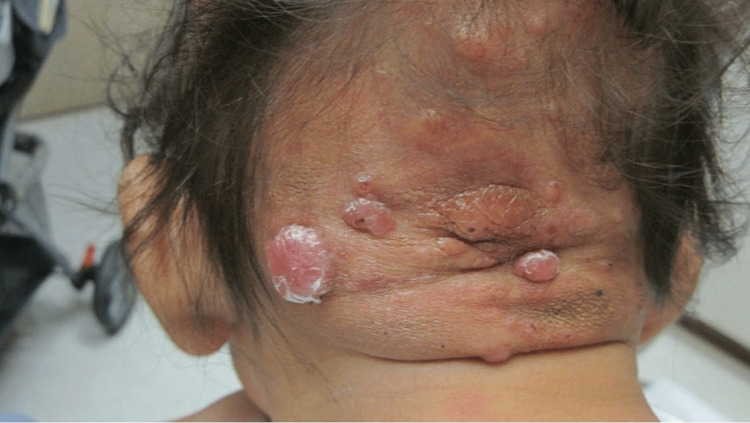
Pale tan-colored pedunculated nodules at seven months of life; left occipital lesion measured 2 cm in diameter.

**Figure 6:**
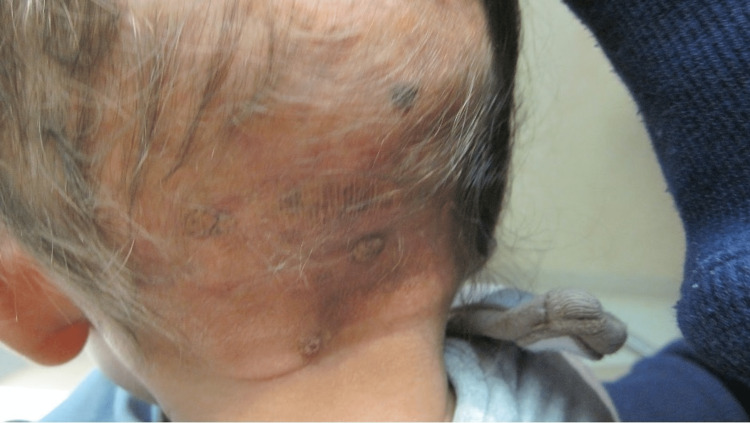
Flat, yellow plaques on the occipital scalp indicative of self-involuted juvenile xanthogranuloma lesions at nine months of age.

Over the course of 23 months, new lesions appeared, grew, shrunk, and self-involuted leaving flat, yellow lesions (Figures 4-6). From the initial presentation of an estimated 1-2 cm in diameter, lesions grew to up to 4.5 cm in diameter, meeting the requirement of >2 cm for the giant variant of JXG (Figure 4). Interestingly, at seven months, some lesions took the form of pale tan-colored pedunculated protrusions, the largest measuring 2 cm in diameter on the left occiput (Figure 5). The two most prominent lesions became a source for trauma and bleeding and were excised. By 23 months of life, all lesions had involuted without treatment and hair growth was normal in all areas of the scalp.

## Discussion

JXG is a benign proliferative disorder of dermal histiocytes that typically presents in early childhood and spontaneously resolves in approximately one to five years [3]. It typically presents as a solitary 0.5-2 cm reddish or yellowish-brown papule, macule, plaque or nodule [1]. Common locations include the head, neck, and upper trunk although they can occur anywhere [1]. Early lesions appear more raised and erythematous before becoming progressively more flat and yellow with lipidation and maturation [4]. Rarely, JXG can present as multiple lesions, giant lesions, or with extracutaneous involvement. Most common extracutaneous sites involved are the liver, lung, spleen, and central nervous system with the most severe cases seen in very young children with multiple skin lesions [2]. In a systematic review of 2949 patients, systemic and ocular involvement with cutaneous JXG occurred in 0.75% and 0.24% respectively [5]. Although fatalities are exceedingly rare, newborns with extensive visceral or ocular involvement typically require aggressive treatment.

Algorithms and classifications have been proposed and used to assist with diagnosing and categorizing JXG [6,7]. Such methods sort JXG presentations by onset, size of lesions, number of lesions, type of lesion, and extra-cutaneous involvement. The giant variant is the rarest subtype of JXG [7] and describes lesions over 2 cm in size [1]. Giant JXG is expected to follow the typical course of JXG with treatment options including observation for self-involution in a few years and surgical excision of symptomatic lesions. A systematic review in 2018 found 51 cases of giant JXG in the English literature, of which 24 (47%) were congenital, six (12%) had multiple lesions, 14 (28%) were nodular, and nine (17%) were nodular-ulcerated [6]. A retrospective study of 76 children with JXG at a children’s hospital in Illinois documented only one case (1.3%) of a mixed macronodular (>1 cm) and micronodular (<1 cm) picture [5]. Furthermore, a retrospective study of 44 children in Turkey classified every patient with multiple JXG lesions (22%) as eruptive rather than congenital [7].

With the background of the current literature, we present a rare case of giant congenital JXG with a mixture of giant and micronodular lesions. This case is also unique in its combination of macules, papules, nodules and ulcerations, some of which were present at birth while others erupted or were acquired throughout infancy until self-involution was complete by 23 months. Additionally, our case documents symptomatic, pale tan-colored pedunculated JXG lesions at seven months of life, the largest of which measured 2 cm.

## Conclusions

To our knowledge, this mixed picture of giant, congenital JXG is the first case of this atypical presentation to be reported and as such, we encourage pediatricians and dermatologists to consider JXG in the differential diagnosis of multiple ulcerated and non-ulcerated macular, papular, and nodular lesions at birth.
